# REFRAMING AGING: THE IMPACT OF LABELS ON ATTITUDES TOWARD OLDER ADULTS

**DOI:** 10.1093/geroni/igae098.0012

**Published:** 2024-12-31

**Authors:** Michael Vale, Ava Bjelka

**Affiliations:** Sacred Heart University, Fairfield, Connecticut, United States; Sacred Heart University, Fairfield, Connecticut, United States

## Abstract

Efforts to combat ageism advocate for avoiding labels, such as “old”, “elderly”, and “senior citizen”; however, these labels persist in everyday speech, and new terms like “Ok Boomer” have recently gained popularity. To examine the perception and impact of such labels on older adults, we had young, middle-aged, and older participants complete an online experiment in which they read a vignette. More specifically, we manipulated the label (i.e., older, old, elderly, senior citizen, Boomer, in their 70s) and gender (i.e., woman, man) of the main character. Preliminary findings from a sample of 512 participants (ages 18-91) were analyzed using 6x2x3 ANOVAs. A three-way interaction was significant for ratings of offensiveness (p<.001, ηP2=.07), with middle-aged participants finding the labels most offensive. Older participants, particularly when the target was described as a woman, were most offended by labels like “elderly” or “old.” Similarly, the appropriateness of labels varied across gender and label types (p=.07, ηP2=.02), with “old” rated more appropriate for women and “Boomer” for men. Differences in labels also affected perceptions of competence (p=.06, ηP2=.02). Meanwhile warmth perceptions varied across age groups (p<.001, ηP2=.11), with young adults viewing targets most warmly. These results suggest that labels matter in shaping perceptions of older adults, especially among the demographic they represent. Our findings can guide initiatives aimed at reframing aging perceptions and highlight how language can shape attitudes towards older adults. The results also underscore how ageism intersects with sexism. Further analysis will be conducted on the full sample (N<900).

